# Parasitic Leiomyoma as a Cause for Primary Small Bowel Obstruction

**DOI:** 10.7759/cureus.23473

**Published:** 2022-03-25

**Authors:** Faseeha Rehman, Samer Talib, Alijandra Razetto, Vasudev Daliparty, Matthew Yotsuya

**Affiliations:** 1 Internal Medicine, Raritan Bay Medical Center, Perth Amboy, USA

**Keywords:** surgical morcellation, meckel's diverticulum, ct scan, blood supply, small bowel obstruction, uterine leiomyoma, parasitic leiomyoma (pl)

## Abstract

Parasitic leiomyomas (PL) are rare cause of small bowel obstruction (SBO) in young women. Usually, they arise in women who underwent laparoscopic or surgical morcellation of uterine fibroids. PL may present with vague abdominal pain, constipation, obstipation, or rarely SBO. SBO can be primary or secondary, depending on prior surgical history. PL might present as primary SBO due to their mass effect or secondary SBO if the patient’s PL resulted from a surgical procedure. We came across a very remarkable presentation of primary SBO due to an artery supplying the PL. Few cases of primary PL have been reported.

## Introduction

Leiomyomas are intraabdominal uterine neoplasms [1]. They are composed of smooth muscle and connective tissue. A vast majority of patients with leiomyomas are asymptomatic. Some can report abnormal uterine bleeding or infertility and pressure on nearby organs with pain. Some report abdominopelvic mass. Leiomyoma is classified into subserosal transmural intramural and submucosal. Subserosal leiomyomas occasionally become parasitic leiomyoma (PL). Pedunculated myoma outgrows its blood supply and adheres to other organs likethe omentum and bowel for blood supply [2]. International Federation of Gynecology and Obstetrics (FIGO) is a classification system for abnormal uterine bleeding founded to help healthcare providers better identify the cause of bleeding and management plans. As per FIGO classification, PL is considered type 8 leiomyoma with no myometrial involvement and no uterine attachment located at ectopic locations like the cervix. PL is either primary spontaneous or secondary. A secondary leiomyoma develops after surgical myomectomy.

## Case presentation

A 59 years old female with a pertinent past medical history of jejunal diverticulitis presented to ED with one day of progressive periumbilical abdominal pain. The pain was 9/10 in intensity sharp intermittent and radiating to the back. The patient denied any aggravating or relieving factors. The patient reported nausea with vomiting and obstipation. The patient vitals are presented in Table 1.

**Table 1:** The vitals of our patient during her presentation to the emergency room. BPM: beats per minute

Blood pressure	142/78 mmHg
Heart Rate	100 BPM
Temperature	97.1°F
Respiratory Rate	21 BPM
SPO_2_	94%
Bodyweight	180 lbs

On the physical exam she appeared lethargic. The abdomen was distended and diffusely tender. The patient’s blood work is presented in Tables 2-3.

**Table 2:** A comprehensive metabolic panel. BUN: blood urea nitrogen; Alk Phos: Alkaline phosphatase; Bili tot: total bilirubin; ALT: alanine transaminase; AST: aspartate transaminase

NA	137 (136-145 mmol/L)
K	3.7 (3.5-5.1 mmol/L)
CL	100 (90-110 mmol/L)
CO_2_	24 (22-28 mmol/L)
BUN	12 (6-20 mg/dL)
Creatinine	0.8 (0.7-1.2 mg/dL)
Glucose	152 (0.7-1.2 mg/dL)
Calcium	10.0 (8.6-10.2 mg/dL)
Alk Phos	102 (40-150 IU/L)
Bili tot	0.4 (0 0.8 mg/dL)
ALT	40 (0-50 IU/L)
AST	40 (10-40 IU/L)
Total protein	6 (60-80 g/dL)

**Table 3:** Complete blood counts.

White blood cells	24.300 (3.6-11 10^3/ µL)
Red blood cells	4.10 (3.80-5.60 10^6/µL)
Hemoglobin	11.5 (11.6-16.8 g/dL)
Hematocrit	35.8 (35.1-50.0%)
Platelet	87.300 (150-372 10^3/µL)

**Figure 1:**
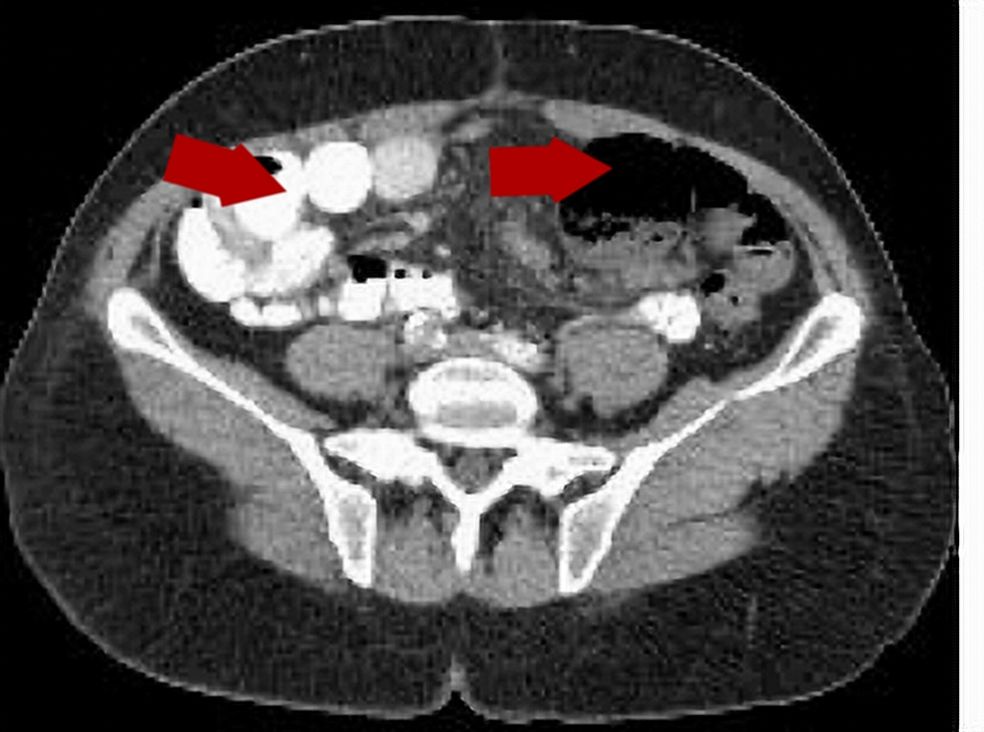
CT scan of abdomen and pelvis revealed focal dilation of mid-jejunal small bowel with inflammatory changes in the mesenteric fat and a jejunal diverticulum.

**Figure 2:**
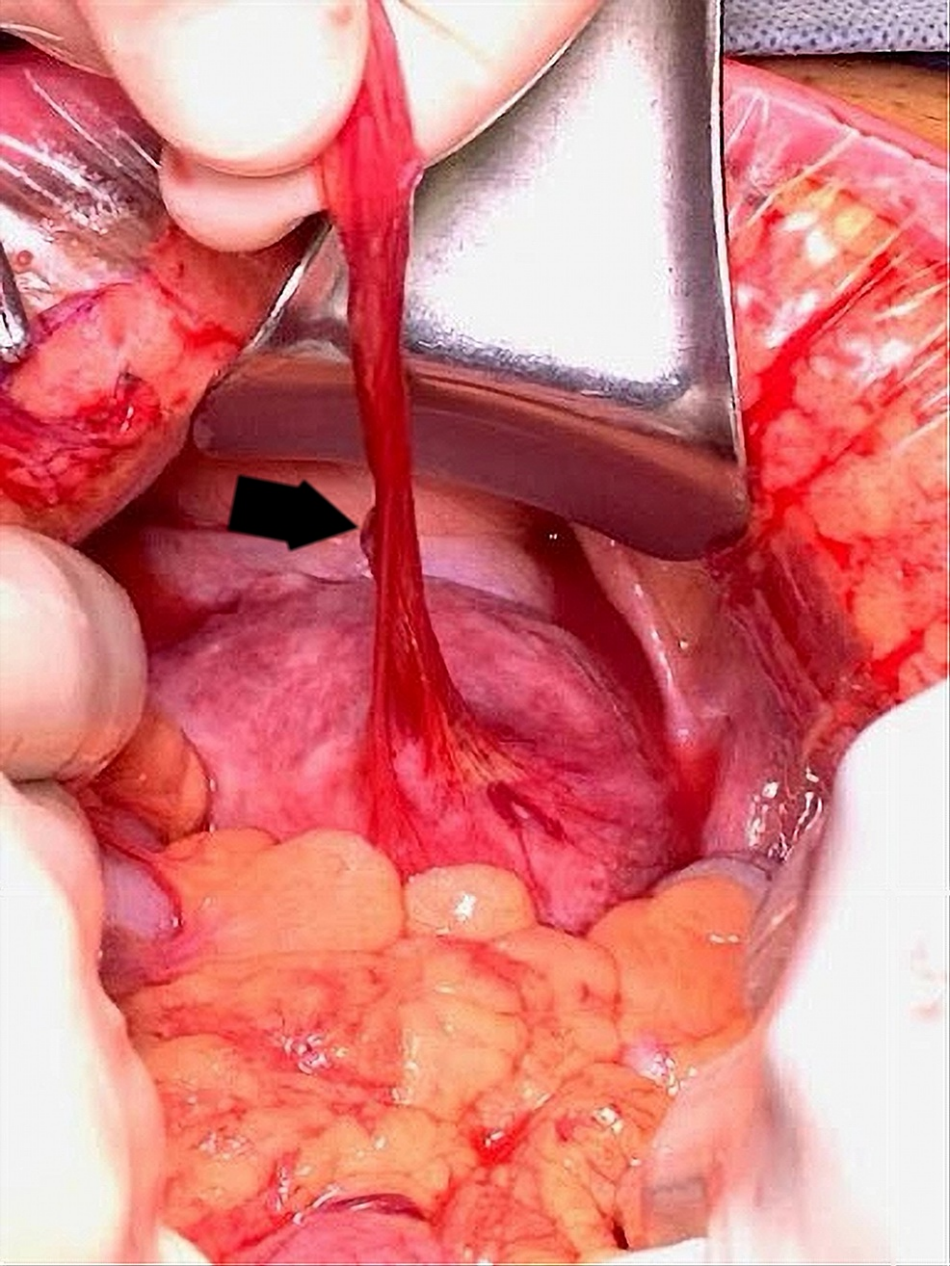
Several blood vessels providing blood supply to the parasitic leiomyoma. The black arrow is pointing toward the blood vessels.

**Figure 3:**
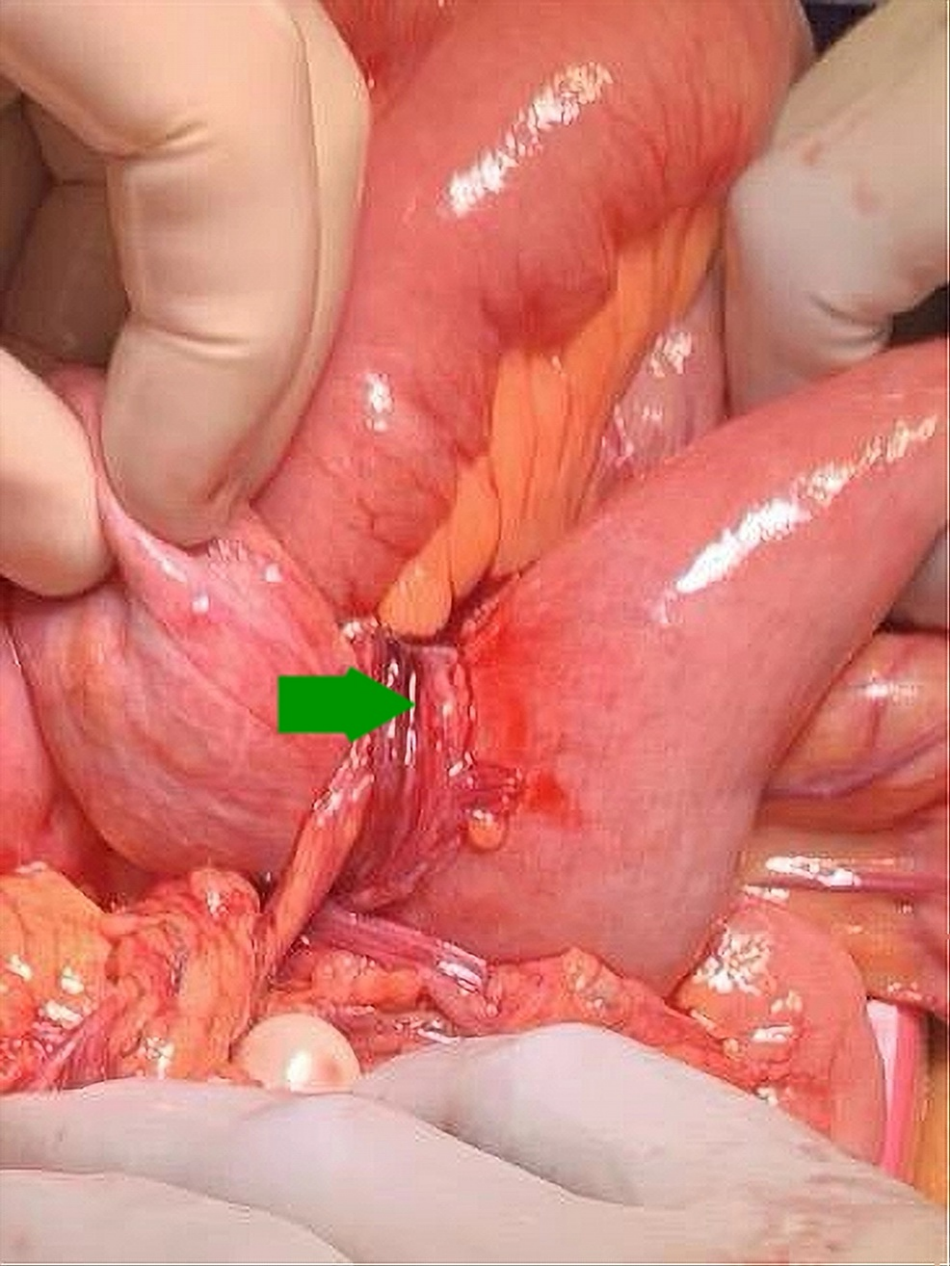
mesenteric vasculature wrapped around the small bowel during its course from the mesentery to the parasitic leiomyoma. The green arrow points to the strangulation site.

**Figure 4:**
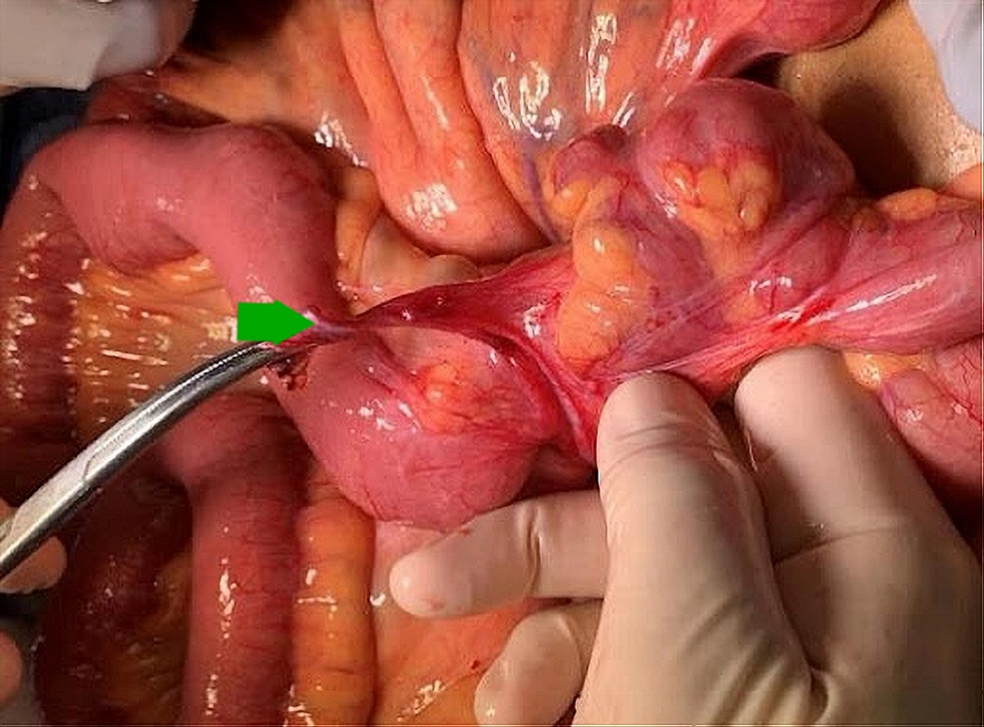
Several additional vessels were also found wrapped around the neck of the patient’s jejunal diverticulum. The green arrow is pointing toward them.

There was a high suspicion of possible small bowel obstruction (SBO), and CT scan of the abdomen and pelvis with contrast showed focal dilation of mid-jejunal small bowel with inflammatory changes in the mesenteric fat and a jejunal diverticulum (Figure 1). Gastroenterology and surgery were consulted. The case was reviewed thoroughly, and the decision to take the patient to the operating room urgently for primary SBO was made. Intraoperative findings were fascinating. The PL in our patient did not cause SBO due to the mass effect but the blood vessels wound around the bowel and caused the bowel obstruction (Figures 2-4). PL derived its blood supply from the mesenteric vasculature to the PL. Blood vessels wrapped around the jejunum and causing a stricture.

The surgeon did a stricturoplasty procedure at the obstruction site with a small bowel resection at the jejunal diverticulum site around 2 cm of the jejunum removed with end-to-end anastomosis. The patient had an uneventful postoperative course. She recovered bowel function and fared well.

## Discussion

Leiomyoma or fibroid are benign tumors of the smooth muscle of the uterus. PL is a rare variant of uterine leiomyoma that grows outside the uterus. FIGO classification of uterine leiomyoma described eight types, providing a representative map of fibroid distributions, according to FIGO, types 2-5 represent submucosal and subserosal leiomyoma while type 8 represents PL. Leiomyoma has a higher incidence in African-American females than Caucasian females. As most patients are asymptomatic, the true incidence/prevalence is difficult to determine [3]. The prevalence is variable among females of different ethnicity and age groups. The prevalence could be as high as 68.6%. It is more prevalent in females in their fourth to sixth decades of life and is associated with early menarche, obesity, and hypertension [4].

PL is considered a subserosal fibroid, while others believe it is an iatrogenic complication of laparoscopic myomectomy or morcellation [5]. PL is a subserosal uterine leiomyoma that survives in the extragenital tract by obtaining blood supply from their surrounding structures and adhering to surrounding structures such as (the broad ligament omentum or retroperitoneal connective tissue). Historically, these masses were thought to occur as pedunculated subserosal uterine fibroids. Either they lose their connection from the uterus and derive blood supply by neovascularization or grow beyond the uterus into the abdomen. This classic theory did not apply to this patient given no past surgical history. PL may generally present with symptoms of mass effect such as chronic abdominal pain with bloating and constipation or nausea.

SBO is a more grave and unusual presentation requiring surgical intervention. The differential diagnosis for PLs includes masses of ovarian origin (primary ovarian neoplasms and metastases) and broad ligament cyst. At the ultrasound, a typical leiomyoma usually has a whorled appearance with variable echogenicity depending on the extent of degeneration and calcification. MRI helps differentiate leiomyomas from solid malignant pelvic tumors. The typical leiomyomas demonstrate low to intermediate signal intensity on T1-weighted images and low signal intensity on T2-weighted images. An ultrasonography-guided biopsy is vital in determining its exact composition before surgery. The PL in our patient did not cause SBO due to the mass effect. It derived its blood supply from the mesenteric vasculature causing strangulation of the jejunum (as described in case presentation). Our patient presented with signs and symptoms of acute abdomen. Our patient did not demonstrate any symptoms suggestive of gynecological etiology for her acute abdomen. SBO is categorized as primary if the patient has no surgical history or secondary bowel obstruction when a patient reports a history of abdominopelvic surgery. In a systematic review and meta-analysis of six studies including 442 patients, the prevalence of neoplastic etiologies for SBO without abdominopelvic surgery history differs from 7.7 to 13.4% at the same time and adhesions were the culprit in 54% of cases [6]. More than half of those patients underwent a non-operative treatment trial often failing. When PLs are diagnosed by imaging and present with symptoms suggestive of fibroid they are treated by laparoscopic surgical removal. However, they warrant surgical intervention if they cause unusual symptoms unrelated to the reproductive system.

## Conclusions

Leiomyomas are common benign tumors of the smooth muscle of the uterus. In rare circumstances, subserosal leiomyoma may detach from the uterus and attach itself to nearby organs. It is called PL. The most typical source of blood supply for the PL is the omentum. PL is a rare type of uterine leiomyoma that affects young women. They have an atypical presentation with symptoms ranging from asymptomatic to mild abdominal pain to a high-grade SBO. Still, the most common symptoms are pressure symptoms, early satiety with bloating during eating, abdominal discomfort, and nausea. Diagnosis is often challenging for clinicians. There is a high index of suspicion for PL in middle-aged women with vague abdominal symptoms. The management is usually through surgery either open or laparoscopic.
